# Pulmonary Artery Size as a Predictor of Early Post-operative Pediatric and Congenital Heart Transplant Outcomes

**DOI:** 10.1007/s00246-025-03839-z

**Published:** 2025-03-27

**Authors:** William A. Harris, Zain S. Kazmi, John M. Costello, Jason R. Buckley, Minoo N. Kavarana, Andrew J. Savage, Varsha M. Bandisode, Anthony M. Hlavacek, Carolyn L. Taylor

**Affiliations:** 1 Division of Pediatric Cardiology, Medical University of South Carolina, Charleston, SC USA; 2 College of Medicine, Medical University of South Carolina, Charleston, SC USA; 3 Division of Pediatric Cardiothoracic Surgery, Medical University of South Carolina, Charleston, SC USA

**Keywords:** Pediatric heart transplant, Acute right ventricular failure, Pulmonary artery size, Heart transplant outcomes

## Abstract

Pulmonary artery size has been studied as an outcome predictor for selected congenital heart surgeries but has not been investigated in pediatric and congenital heart transplantation. We sought to evaluate pre-operative pulmonary artery size as a predictor of post-transplant outcomes. This single center retrospective study included all patients transplanted from 2014 to 2023. Echocardiography, computed tomography angiography (CTA), and catheter angiography were used to measure pre-operative pulmonary artery size. Cross-sectional areas were calculated and indexed to calculate Nakata index and lower lobe index. Outcomes included post-transplant cardiovascular and end-organ function, length of stay, and mortality. Statistical analyses included Spearman rank correlations, chi-squared tests, and Mann–Whitney *U* tests. Included were 68 patients with 37 (54%) having a diagnosis of congenital heart disease. Echocardiogram (*n* = 68), CTA (*n* = 52), and catheter angiogram (*n* = 31) measurements were analyzed. Smaller lower lobe index was associated with longer duration of post-transplant treatment with nitric oxide (*p* = 0.02). In the congenital heart disease cohort, smaller Nakata index was associated with pulmonary artery reconstruction during transplant (*p* = 0.003). Indexed pulmonary artery size was otherwise not associated with important outcomes in the entire population or congenital heart disease cohort. While pre-operative pulmonary artery size was associated with prolonged nitric oxide use, it was generally not predictive of other early post-transplant outcomes. No imaging modality was found to have superior predictive value. Use of advanced imaging to guide surgical technique during transplant may negate the impact of smaller pulmonary artery size.

## Introduction

Although post-operative survival in pediatric orthotopic heart transplantation (OHT) continues to improve, acute right ventricular (RV) failure remains an important cause of early morbidity [1–3]. Previous studies have identified early RV failure in 9–25% of pediatric recipients. Several predictors for RV failure have been identified, including congenital heart disease (CHD), pre-OHT pulmonary vascular resistance (PVR), pre-OHT ventilator support, and prolonged donor ischemic time [3–5]. Further investigations into pre-operative factors to identify patients at high risk for post-OHT graft and isolated RV failure are ongoing [5, 6].

Pulmonary artery (PA) size has been studied as a predictor of outcomes in single ventricle palliation patients, but there are no published studies evaluating PA size as a predictor of post-OHT outcomes [7–10]. Several measurements of PA size have been investigated in single ventricle literature. Among the most studied are the Nakata index (NI), which is a ratio of proximal branch PA cross-sectional areas indexed to body surface area (BSA), and the lower lobe index (LLI), which is a BSA-indexed ratio of the lower lobe branch PA cross-sectional areas. The LLI may represent a more accurate measurement of PA size and growth in repaired CHD patients given the frequency of more proximal branch PA reconstruction during staged palliation surgeries [11, 12].

These PA measurements and related indices are readily obtained by echocardiography, computed tomography angiography (CTA), and catheterization angiography. Our aims with this study were to determine whether PA size has any predictive value for outcomes in the early post-OHT period, particularly with regards to acute RV failure. We hypothesized that smaller PA size would be associated with worse outcomes, particularly in patients with CHD. Furthermore, we sought to investigate various imaging modalities used to measure PA size to determine if any of these is more predictive.

## Patients and Methods

### Study Design and Population

We conducted a single center retrospective study of all pediatric and congenital patients who underwent OHT at the Medical University of South Carolina (MUSC) from July 2014 to December 2023. There were no specific exclusion criteria. The study protocol was approved by the Institutional Review Board at MUSC and the need for written informed consent was waived. Patient charts were reviewed electronically through Epic (Epic Systems Corporation, Verona, WI).

### Pre-OHT Data and Pulmonary Artery Measurements

Primary cardiac diagnosis was identified as CHD, dilated cardiomyopathy (DCM), restrictive cardiomyopathy (RCM), or myocarditis. CHD diagnosis was further categorized by stage of single ventricle palliation where applicable. Duration of pre-OHT mechanical or inotropic support was recorded. Clinical markers of pre-OHT status were recorded including baseline oxygen saturation, end-organ dysfunction as defined by baseline creatinine greater than 2.0 mg/dL and/or total bilirubin greater than 2.0 mg/dL, and growth failure as defined by weight below the fifth percentile for age [13]. Pre-operative invasive catheter hemodynamics were recorded including BSA-indexed PVR (WU*m^2^) [3].

Pre-operative echocardiograms, CTA, and catheter angiograms which were acquired as a part of routine clinical care closest to OHT were reviewed. In studies with adequate PA images, the investigators with relevant expertise in each modality confirmed the following measurements. Branch PAs were measured immediately distal to the bifurcation from the main PA and *z*-scores were recorded (Boston Children’s Hospital *z*-score calculator). Cross-sectional areas were calculated using the PA measurement as the diameter of an assumed circular cross-section. The NI was calculated by adding the branch PA cross-sectional areas and dividing by the BSA (m^2^) [11]. On CTA and catheter angiograms, the lower lobe branch PAs were measured. Lower lobe cross-sectional areas were calculated and divided by BSA to obtain the LLI [7].

### Surgical and Post-Operative Data

Surgical data including donor ischemic time and additional procedures during OHT were recorded. Post-OHT data collection included ICU and total length of stay (LOS), 24 h maximum vasoactive-inotropic score (VIS) [14], duration of ICU support with inotropes, inhaled nitric oxide and mechanical ventilation, and characterization of end-organ function. Post-operative acute kidney injury (AKI) was quantified by the ratio of peak creatinine within the first post-operative week to baseline pre-OHT creatinine, which was used to calculate AKI stage following KDIGO guidelines [15]. Post-operative RV function was assessed with hemodynamic trends on ICU monitoring data, echocardiogram measurements at several timepoints, and catheter measurements at one-month post-OHT. Per prior studies, clinical evidence of acute RV failure was defined as post-operative CVP > 16 mmHg and evidence of end-organ dysfunction (total bilirubin > 2.0 mg/dL, creatinine > 2.0 mg/dL, or dialysis); echocardiographic evidence of acute RV dysfunction was defined as qualitatively moderately-to-severely depressed RV function or moderate-to-severe tricuspid regurgitation on the initial post-operative transthoracic echocardiogram obtained at post-operative day #1-3 [3, 13, 16].

### Statistical Analyses

Descriptive statistics were generated for the study population. Continuous variables were reported with medians and interquartile ranges. Categorical variables were reported with counts and percentages. Comparisons of CHD and non-CHD patient cohorts were conducted with Pearson’s Chi-squared tests, Fisher’s exact tests, and Mann–Whitney *U* tests. Comparisons of PA size measurements versus post-operative outcomes were conducted with Spearman’s rank correlations and Mann–Whitney *U* tests. A *p* value of less than 0.05 was considered statistically significant. Analyses were performed with Excel (Microsoft Corporation, Redmond WA) and Statistical Product and Service Solutions (SPSS) version 25 (IBM, Armonk, NY).

## Results

### Demographic and Clinical Data

Sixty-eight patients underwent OHT during the study period. No patients who underwent OHT during the period were excluded. Thirty-seven patients (54%) had a primary diagnosis of CHD. Of the CHD patients, the majority (24, 65%) had previously undergone Fontan palliation. Most non-CHD patients had a primary diagnosis of DCM (22 of 31, 71%). CHD patients were older at OHT than non-CHD patients (median age 14.0 years vs 4.8 years, *p* = 0.015) but there were no other significant demographic differences between cohorts. CHD patients had lower baseline oxygen saturation (median 89% vs 97%, *p* < 0.001) and higher baseline total bilirubin (0.9 mg/dL vs 0.5 mg/dL, *p* < 0.001) and creatinine (0.7 mg/dL vs 0.5 mg/dL, *p* = 0.011) than non-CHD patients. Non-CHD patients were more likely to be on mechanical circulatory support at the time of OHT than CHD patients (81% vs 27%, *p* < 0.001). Non-CHD patients also had higher indexed pulmonary vascular resistance (3.6 WU*m^2^ vs 2.4 WU*m^2^, *p* = 0.018) on pre-OHT invasive hemodynamics than CHD patients (Table 1).

**Table 1:** Demographics and pre-OHT characteristics

	All patients (*n* = 68)	CHD (*n* = 37)	Non-CHD (*n* = 31)	*p*
Age at OHT (years)	11.1 (2.3, 15.8)	14.0 (5.9, 16.9)	5.0 (1.0, 15.3)	0.02*
Weight (kg)	30.8 (12.3, 58.4)	44.2 (18.6, 59.0)	17.5 (8.2, 58.8)	0.16
BSA (m^2^)	1.10 (0.51, 1.63)	1.36 (0.72, 1.62)	0.67 (0.40, 1.67)	0.16
Male sex	44 (65%)	27 (73%)	17 (55%)	0.12
White race	35 (51%)	22 (60%)	12 (39%)	0.09
Non-Hispanic ethnicity	64 (94%)	34 (92%)	30 (97%)	0.62
Specific diagnosis				
Fontan	24 (35%)	24 (65%)	–	
Bidirectional Glenn	4 (6%)	4 (11%)	–	
DCM	22 (32%)	–	22 (71%)	
RCM	3 (4%)	–	3 (10%)	
Myocarditis	4 (6%)	–	4 (13%)	
Other	11 (16%)	9 (24%)	2 (6%)	
Mechanical support	35 (52%)	10 (27%)	25 (81%)	< 0.001*
Type of mechanical support				
VAD	30 (44%)	8 (22%)	22 (71%)	
BiVAD	4 (6%)	1 (3%)	3 (10%)	
ECMO	3 (4%)	1 (3%)	2 (6%)	
Duration of mechanical support (days)	96 (54, 176)	73 (36, 265)	99 (64, 170)	0.73
Inotropic support	55 (81%)	33 (89%)	22 (71%)	0.06
Duration of inotropic support (days)	149 (55, 271)	139 (43, 363)	155 (91, 207)	0.67
Ventilator support at time of OHT	4 (6%)	3 (8%)	1 (3%)	0.62
Inpatient prior to transplant	53 (78%)	29 (78%)	24 (77%)	0.92
Baseline systemic O_2_ saturation (%)	97 (87, 99)	89 (84, 97)	99 (97, 100)	< 0.001*
Baseline total bilirubin (mg/dL)	0.6 (0.4, 1.0)	0.9 (0.6, 1.6)	0.5 (0.3, 0.6)	< 0.001*
Baseline creatinine (mg/dL)	0.5 (0.4, 0.8)	0.7 (0.5, 0.8)	0.5 (0.3, 0.6)	0.01*
Pre-OHT cath	60 (88%)	35 (95%)	25 (81%)	0.04*
Pre-OHT PVRi (WU*m^2^)	2.9 (2.0, 4.1)	2.4 (1.9, 3.4)	3.6 (2.4, 4.4)	0.02*

Data are presented as median (interquartile range) or count (percent)

*BiVAD* biventricular assist device, *BSA* body surface area, *DCM* dilated cardiomyopathy, *ECMO* extracorporeal membrane oxygenation, *VAD* ventricular assist device, *OHT* orthotopic heart transplant, *PVRi* pulmonary vascular resistance (indexed), *RCM* restrictive cardiomyopathy

Bolding and asterisk (*) were used to visually signify a significant *p* value < 0.05

From a surgical standpoint, the donor ischemic time was longer for CHD patients (301 min vs 240 min, *p* < 0.001), and CHD patients were more likely to undergo PA reconstruction during OHT (78% vs 0%, *p* < 0.001). Post-operatively, CHD patients spent longer on ventilator support (4 days vs 2 days, *p* < 0.001) and inhaled nitric oxide (3 days vs 1 day, *p* = 0.003) than non-CHD patients. CHD patients also had higher peak total bilirubin (3.4 mg/dL vs 1.5 mg/dL, *p* < 0.001) and were more likely to require dialysis (32% vs 3%, *p* = 0.004) after OHT than non-CHD patients. CHD patients were more likely to meet criteria for acute RV failure post-OHT (27% vs 6%, *p* = 0.027). There were two deaths within 30 days of transplant, both in CHD patients (Table 2).

**Table 2:** Surgical and post-OHT characteristics

	All patients	CHD	Non-CHD	*p*
	(*n* = 68)	(*n* = 37)	(*n* = 31)	
Surgical data				
Donor ischemic time (min)	255 (222, 308)	301 (238, 337)	240 (220, 256)	< 0.001*
PA reconstruction	29 (43%)	29 (78%)	0 (0%)	< 0.001*
Other surgical procedures during OHT	41 (60%)	16 (43%)	25 (81%)	0.002*
Other procedure type				
VAD explant	35 (51%)	10 (27%)	25 (81%)	
ECMO cannulation	4 (6%)	4 (11%)	0 (0%)	
Liver transplant	1 (1%)	1 (3%)	0 (0%)	
Post-operative data				
Post-op ICU days	8 (6, 15)	10 (7, 19)	8 (5, 13)	0.13
Post-op hospital days	17 (11, 30)	17 (12, 32)	16 (11, 28)	0.44
30-day mortality	2 (3%)	2 (5%)	0 (0%)	0.5
Max vasoactive-inotropic score	12.5 (10, 15.7)	12.5 (10.8, 18.0)	12.5 (10.0, 13.5)	0.35
Days on inotropic support	9 (6, 26)	11 (7, 28)	9 (5, 25)	0.43
Days of ventilator support	3 (2, 5)	4 (2, 11)	2 (1, 3)	0.001*
Days on nitric oxide	2 (1, 4)	3 (1, 7)	1 (1, 3)	0.003*
Peak total bilirubin (mg/dL)	2.4 (1.2, 3.8)	3.4 (2.0, 5.1)	1.5 (0.8, 2.5)	< 0.001*
Creatinine ratio, peak to preoperative baseline	1.6 (1.3, 2.4)	1.7 (1.3, 3.0)	1.6 (1.3, 2.0)	0.78
Use of dialysis	13 (19%)	12 (32%)	1 (3%)	0.002*
Mean CVP POD#2 (mmHg)	13 (10, 15)	13 (11, 16)	12 (11, 15)	0.36
Clinical evidence of RVF	12 (18%)	10 (27%)	2 (6%)	0.03*
Moderate or severe RV dysfunction POD#1–3	15 (22%)	7 (19%)	8 (26%)	0.49
Moderate or severe TR POD#1–3	5 (7%)	4 (11%)	1 (3%)	0.23

Data are presented as median (interquartile range) or count (percent)

*CVP* central venous pressure, *ECMO* extracorporeal membrane oxygenation, *LOS* length of stay, *OHT* orthotopic heart transplant, *PA* pulmonary artery, *POD* post-operative day, *RVF* right ventricular failure, *TR* tricuspid regurgitation, *VAD* ventricular assist device

Bolding and asterisk (*) were used to visually signify a significant *p* value < 0.05

### Analysis of Pulmonary Artery Size

All patients in the study population had pre-OHT echocardiograms. CHD patients had significantly lower NI (137 mm^2^/m^2^ vs 190 mm^2^/m^2^, *p* = 0.021) by echocardiogram than non-CHD patients. Most patients in both cohorts (86% for CHD, 65% for non-CHD) had pre-OHT CTA with no significant differences in PA measurements between cohorts. Thirty-one of the CHD patients (83%) had catheter PA angiography prior to OHT; none of the non-CHD patients had pre-OHT catheter PA angiography (Table 3).

**Table 3:** Pulmonary artery measurements and indices

	Total cohort (*n* = 68)	Primary diagnosis CHD (*n* = 37)	Primary diagnosis non-CHD (*n* = 31)	*p* value
Echocardiography				
Echo prior to OHT, *n* (%)	68 (100)	37 (100)	31 (100)	–
RPA dimension (cm)	1.0 (0.9, 1.3)	1.0 (0.8, 1.3)	1.0 (0.8, 1.2)	0.45
RPA *z*-score	− 0.2 (− 1.2, 0.7)	− 0.2 (− 1.5, 0.3)	− 0.3 (− 1.0, 0.8)	0.39
LPA dimension (cm)	1.0 (0.8, 1.2)	1.0 (0.8, 1.2)	0.9 (0.8, 1.3)	0.93
LPA *z*-score	− 0.1 (− 1.2, 0.8)	− 0.6 (− 1.4, 0.6)	0.3 (− 0.5, 1.0)	0.03*
Nakata index (mm^2^/m^2^)	176 (119, 219)	137 (95, 206)	190 (160, 234)	0.02*
CT angiography				
CTA prior to OHT, *n* (%)	52 (76)	32 (86)	20 (65)	–
Proximal RPA dimension (mm)	12.9 (9.5, 16.7)	13.0 (10.2, 16.9)	12.1 (8.3, 16.1)	0.29
RPA *z*-score	0.8 (− 0.2, 1.7)	1.1 (− 0.1, 1.8)	0.6 (− 0.3, 1.1)	0.42
Proximal LPA dimension (mm)	11.8 (8.4, 15.8)	11.6 (8.6, 15.2)	12.5 (7.1, 16.2)	0.64
LPA *z*-score	0.2 (− 0.8, 1.5)	0.1 (− 1.0, 1.2)	0.6 (− 0.3, 1.5)	0.10
Nakata index (mm^2^/m^2^)	240 (188, 319)	247 (181, 299)	224 (204, 306)	0.81
Right lower lobe PA dimension (mm)	10.9 (7.4, 12.9)	11.4 (8.5, 12.9)	9.0 (5.1, 12.1)	0.11
Left lower lobe PA dimension (mm)	9.8 (8.1, 13.4)	9.8 (8.3, 12.8)	9.5 (5.2, 13.7)	0.52
Lower lobe index (mm^2^/m^2^)	161 (133, 200)	167 (140, 203)	140 (119, 191)	0.17
Catheter angiography				
Cath angios prior to OHT, *n* (%)	31 (45)	31 (83)	0 (0)	–
Proximal RPA dimension (mm)	13.8 (9.3, 16.9)	13.8 (9.3, 16.9)	–	–
RPA *z*-score	0.9 (− 0.5, 1.9)	0.9 (− 0.5, 1.9)	–	–
Proximal LPA dimension (mm)	10.4 (7.7, 13.4)	10.4 (7.7, 13.4)	–	–
LPA *z*-score	− 0.6 (− 2.0, 0.6)	− 0.6 (− 2.0, 0.6)	–	–
Nakata index (mm^2^/m^2^)	196 (154, 281)	196 (154, 281)	–	–
Left lower lobe PA dimension (mm)	9.6 (6.5, 11.8)	9.6 (6.5, 11.8)	–	–
Right lower lobe PA dimension (mm)	10.6 (7.4, 13)	10.6 (7.4, 13)	–	–
Lower lobe index (mm^2^/m^2^)	148 (100, 184)	148 (100, 184)	–	–

Data are presented as median (interquartile range) or count (percent)

*CTA* computerized tomography angiography, *LPA* left pulmonary artery, *OHT* orthotopic heart transplant, *RPA* right pulmonary artery

Bolding and asterisk (*) were used to visually signify a significant *p* value < 0.05

For the entire population, patients with a smaller NI by echocardiogram had a higher peak total bilirubin after OHT (*r* =  − 0.306, *p* = 0.011). Otherwise, there were no significant associations between indexed PA size by any modality and post-OHT outcomes including ICU or total hospital LOS, maximum 24 h VIS, days on nitric oxide, or ventilator days. In the CHD cohort, patients with a smaller LLI as measured by catheter angiography spent longer on inhaled nitric oxide post-OHT (*r* =  − 0.416, *p* = 0.020; Table 4). For the entire population and the CHD cohort, there were no significant associations between indexed PA size and post-operative acute clinical RV failure. CHD patients who developed post-OHT acute kidney injury had smaller NI by CTA (232 mm^2^/m^2^ vs 300 mm^2^/m^2^, *p* = 0.03; Table 5).

**Table 4:** Associations between PA size and continuous post-OHT outcome measures

		Max VIS first 24 h	Days on nitric oxide	Days on ventilator	Peak total bilirubin	ICU LOS	Hospital LOS
All patients							
Echo NI	*r*	0.146	− 0.174	− 0.001	− **0.306**	0.106	0.163
	*p*	0.23	0.16	0.99	0.01*	0.4	0.2
CTA NI	*r*	0.018	0.01	− 0.006	0.044	0.082	0.001
	*p*	0.9	0.94	0.96	0.75	0.58	0.99
CTA LLI	*r*	0.121	0.015	0.108	0.131	0.152	0.06
	*p*	0.4	0.92	0.45	0.36	0.31	0.69
CHD cohort							
Echo NI	*r*	0.104	− 0.192	− 0.014	− 0.068	0.046	0.056
	*p*	0.54	0.26	0.93	0.69	0.8	0.76
CTA NI	*r*	0.016	− 0.237	− 0.143	0.072	0.026	− 0.06
	*p*	0.93	0.19	0.43	0.7	0.9	0.76
CTA LLI	*r*	0.113	− 0.347	− 0.079	− 0.013	0.173	0.069
	*p*	0.54	0.06	0.67	0.94	0.39	0.73
Cath NI	*r*	− 0.103	− 0.307	− 0.281	− 0.203	− 0.154	− 0.167
	*p*	0.58	0.09	0.24	0.27	0.44	0.41
Cath LLI	*r*	0.054	− **0.416**	− 0.206	− 0.069	0.011	− 0.132
	*p*	0.78	0.02*	0.27	0.71	0.96	0.51

Data presented are Spearman correlation coefficients

*CHD* congenital heart disease, *CTA* computerized tomography angiography, *ICU* intensive care unit, *LLI* lower lobe index, *LOS* length of stay, *NI* Nakata index, *VIS* vasoactive-inotropic score

Bolding and asterisk (*) were used to visually signify a significant *p* value < 0.05

**Table 5:** Associations between PA size and post-OHT end-organ and RV function

	All patients (*n* = 68)		*p*
	AKI (*n* = 48)	No AKI (*n* = 20)	
Echo NI	176 (112, 224)	183 (135, 219)	0.40
CTA NI	232 (178, 311)	264 (212, 341)	0.36
CTA LLI	161 (124, 200)	167 (136, 204)	0.71

	Dialysis (*n* = 13)	No dialysis (*n* = 55)	
Echo NI	138 (105, 190)	186 (123, 229)	0.19
CTA NI	256 (185, 297)	235 (188, 337)	0.81
CTA LLI	185 (145, 198)	160 (132, 203)	0.60

	Clinical RVF (*n* = 12)	No clinical RVF (*n* = 56)	
Echo NI	176 (95, 206)	174 (125, 227)	0.68
CTA NI	261 (176, 304)	235 (191, 330)	0.89
CTA LLI	167 (98, 127)	160 (134, 201)	0.76

	Echo RV dysfunction (*n* = 18)	No echo RV dysfunction (*n* = 50)	
Echo NI	181 (116, 236)	174 (120, 221)	0.62
CTA NI	253 (201, 354)	232 (178, 289)	0.28
CTA LLI	167 (134, 223)	161 (132, 196)	0.66

CHD Cohort (*n* = 37)			
	AKI (*n* = 28)	No AKI (*n* = 9)	
Echo NI	137 (94, 206)	139 (126, 208)	0.77
CTA NI	232 (165, 289)	300 (212, 237)	0.03*
CTA LLI	160 (139, 197)	179 (167, 308)	0.18
Cath NI	177 (136, 302)	207 (191, 240)	0.44
Cath LLI	131 (90, 191)	141 (128, 180)	0.61

	Dialysis (*n* = 12)	No dialysis (*n* = 25)	
Echo NI	137 (101, 197)	139 (92, 213)	0.96
CTA NI	269 (179, 297)	237 (181, 313)	0.94
CTA LLI	188 (153, 202)	161 (137, 205)	0.52
Cath NI	167 (147, 268)	206 (169, 292)	0.25
Cath LLI	127 (100, 198)	146 (123, 184)	0.60

	Clinical RVF (*n* = 10)	No clinical RVF (*n* = 27)	
Echo NI	190 (96, 213)	137 (94, 200)	0.41
CTA NI	279 (166, 311)	237 (179, 300)	0.74
CTA LLI	180 (131, 211)	167 (141, 204)	0.77
Cath NI	165 (143, 370)	207 (166, 287)	0.56
Cath LLI	129 (107, 280)	141 (119, 186)	1.00

	Echo RV dysfunction (*n* = 10)	No echo RV dysfunction (*n* = 27)	
Echo NI	156 (109, 210)	137 (94, 206)	0.51
CTA NI	270 (183, 349)	244 (179, 286)	0.48
CTA LLI	167 (140, 297)	175 (144, 199)	0.98
Cath NI	181 (152, 297)	199 (121, 186)	0.98
Cath LLI	129 (100, 178)	154 (121, 186)	0.51

Data are presented as median (interquartile range). PA indices reported in mm^2^/m^2^

*AKI* acute kidney injury, *CTA* computerized tomography angiography, *LLI* lower lobe index, *NI* Nakata index, *OHT* orthotopic heart transplant, *RVF* right ventricular failure

Bolding and asterisk (*) were used to visually signify a significant *p* value < 0.05

Smaller NI by echocardiogram was associated with higher tricuspid annular plane systolic excursion (TAPSE) on surveillance echocardiography post-OHT. Smaller LLI by CTA was associated with more severe tricuspid regurgitation (Table 6). Smaller NI by echocardiogram was associated with higher mean right atrial pressure, RV end-diastolic pressure, and pulmonary capillary wedge pressure, and (paradoxically) lower PVR on catheterization at one-month post-OHT (Table 7).

**Table 6:** Associations between PA size and post-OHT echocardiography measurements

	TR Severity	RV FAC	TAPSE	TV S′
1–3 days post-OHT				
Echo NI				
*r*	–	− 0.027	− **0.316**	− 0.244
*p*	0.44	0.87	0.03*	0.2
CTA NI				
*r*	–	0.081	− 0.094	0.152
*p*	0.2	0.71	0.59	0.52
CTA LLI				
*r*	**–**	0.344	− 0.308	0.047
*p*	0.67	0.11	0.07	0.84
5–10 days post-OHT				
Echo NI				
*r*	–	0.106	− 0.258	− 0.107
*p*	0.95	0.49	0.06	0.57
CTA NI				
*r*	–	− 0.198	0.201	0.021
*p*	0.68	0.3	0.22	0.92
CTA LLI				
*r*	–	− 0.201	0.19	0.021
*p*	0.07	0.31	0.25	0.92

Data presented are *p* values from Mann–Whitney *U* tests (TR) and Spearman correlations (RV FAC, TAPSE, TV S’). TR severity graded as none-to-mild or moderate-to-severe

*CTA* computerized tomography angiography, *LLI* lower lobe index, *NI* Nakata index, *OHT* orthotopic heart transplant, *RV* right ventricle, *TAPSE* tricuspid annular plane systolic excursion, *TR* tricuspid regurgitation

Bolding and asterisk (*) were used to visually signify a significant *p* value < 0.05

**Table 7:** Associations between PA size and continuous post-OHT catheter hemodynamics

	Mean RAP	RV EDP	Mean PAP	PCWP	PVR
1 Month post-OHT					
Echo NI					
*r*	− **0.25**	− **0.26**	− 0.138	− **0.431**	**0.287**
*p*	0.05*	0.04*	0.28	< 0.001*	0.02*
CTA NI					
*r*	0.019	− 0.001	− 0.031	− 0.122	0.026
*p*	0.9	0.99	0.84	0.41	0.86
CTA LLI					
*r*	0.137	0.133	0.211	0.164	− 0.001
*p*	0.36	0.38	0.16	0.28	0.99

Data presented are Spearman correlation coefficients

*CTA* computerized tomography angiography, *EDP* end-diastolic pressure, *LLI* lower lobe index, *NI* Nakata index, *OHT* orthotopic heart transplant, *PAP* pulmonary artery pressure, *PCWP* pulmonary capillary wedge pressure, *PVR* pulmonary vascular resistance, *RAP* right atrial pressure, *RV* right ventricle

Bolding and asterisk (*) were used to visually signify a significant *p* value < 0.05

CHD patients with smaller NI by echocardiogram were more likely to have their PAs reconstructed during OHT (134 mm^2^/m^2^ vs 259 mm^2^/m^2^, *p* = 0.003). Patients with CHD who underwent PA reconstruction during OHT had a lower max VIS (12 vs 18, *p* = 0.032) within the first 24 h post-OHT than CHD patients who did not undergo PA reconstruction; otherwise outcomes were similar (Table 8). The two patients who suffered early mortality had echocardiographic NI of 232 mm^2^/m^2^ and 137 mm^2^/m^2^ relative to a median index of 137 mm^2^/m^2^ for the CHD cohort; both underwent PA reconstruction during OHT.

**Table 8:** Associations between PA reconstruction and post-OHT outcomes in CHD cohort

	PA reconstruction (*n* = 29)	No PA reconstruction (*n* = 8)	*p*
Max VIS within first 24 h	12 (10, 15)	18 (14, 25)	0.03*
Days on nitric oxide	3 (1, 7)	3 (1, 7)	0.96
Days on ventilator	4 (2, 11)	5 (3, 13)	0.57
ICU LOS (days)	10 (7, 16)	13 (7, 24)	0.25
Hospital LOS (days)	13 (11, 28)	28 (15, 41)	0.16
Peak total bilirubin (mg/dL)	3.7 (2.7, 5.5)	2.4 (0.5, 4.2)	0.06
Acute kidney injury	21 (72%)	7 (88%)	0.65
Post-OHT dialysis	11 (38%)	1 (13%)	0.23
Clinical RVF	8 (28%)	2 (25%)	1.00
Echo RV dysfunction	8 (28%)	2 (25%)	1.00

Data are presented as median (interquartile range) or count (percent). Statistics are *p* values from Chi-square and Mann–Whitney *U* tests

*ICU* intensive care unit, *LOS* length of stay, *OHT* orthotopic heart transplant, *PA* pulmonary artery, *POD* post-operative day, *RVF* right ventricular failure, *VIS* vasoactive-inotropic score

Bolding and asterisk (*) were used to visually signify a significant *p* value < 0.05

## Discussion

The principal finding of our study is that PA size was not significantly associated with important post-OHT outcomes including acute RV failure in our cohort. Our cohort had an overall 30-day survival rate of 97% (66/68) with 18% (12/68) developing clinical evidence of acute RV failure; both are favorable results compared with recent literature.

### Comparison of Our Cohort with Other Studies

Our cohort resembles other single center and multicenter populations in the literature with regards to primary diagnosis and other pre-OHT factors. Other studies have previously described increased baseline organ dysfunction in CHD patients and higher likelihood of pre-OHT mechanical support in DCM patients as seen in our population [2, 4, 5, 17]. Our cohort had higher rates of pre-OHT inotropic (81% vs 50%) and mechanical support (52% vs 20%) but were hospitalized at a similar rate (78% vs 72%) compared to recent ISHLT data as reported by Singh et al. [1].

The incidence of post-OHT acute RV failure was lower in our overall (18% vs 25%) and CHD (27% vs 41%) cohort as compared to the population reported by Hoskote et al. but similar to that observed by Murtuza et al. (18% vs 21% for total cohort, 27% vs 26% for CHD population). We found a similar association between a primary diagnosis of CHD and post-OHT RV dysfunction as reported in both of those studies [3, 16].

### Prior Investigations of Pulmonary Artery Size

PA size has been studied as both a consequence of and an outcome predictor in CHD surgeries, and this work serves as a foundation for our research question. Poor to absent PA growth following the bidirectional Glenn and Fontan procedures of single ventricle palliation has been well described [10, 12, 18]. In 1984, Nakata et al. reported increased mortality in several subgroups of post-surgical CHD patients with smaller PAs and proposed cutoff PA indices for surgical candidacy [11]. In 2007, Adachi et al. found no significant associations between smaller PA indices and midterm mortality or morbidity after Fontan, which the authors felt was reflective of evolving surgical technique [18]. More recently, smaller PAs have been associated with worsening functional status over long-term follow up in Fontan patients [9, 10]. While the threats leading to suboptimal late outcomes in palliated single ventricle patients may differ from those facing OHT recipients, the impact of PA size on pre-OHT heart failure or post-OHT outcomes has not been directly studied. In OHT literature, Chen et al. reviewed 20 years of CHD patients undergoing OHT at their center and identified a negative association between intraoperative PA reconstruction and post-OHT survival but did not specifically investigate PA size [19].

### Associations with Pulmonary Artery Size and Outcomes in Our Cohort

We pursued this investigation as a part of ongoing efforts to further stratify patients known to be at higher risk for adverse post-OHT outcomes, particularly the Fontan population. As established above, palliated single ventricle patients have suboptimal PA growth as a result of passive cavopulmonary blood flow and smaller PAs may impact their short- and long-term outcomes. While the passive pulmonary circulation associated with cavopulmonary connections inherently differs from the right-sided heart function concerns following OHT, the general principle that both patient groups may have better outcomes with a favorable pulmonary vascular bed served as an important nidus for conducting this study. Specifically, we identified PA size as a potential variable of interest in post-OHT outcomes, as donor right ventricles are typically accustomed to facing low afterload yet may need to pump into an unfavorable pulmonary vascular bed in recipients. While the proximal branch PAs are often reconstructed during OHT, there is little to be done operatively to address the overall pulmonary vascular bed. The NI and LLI, while imperfect, offer quantitative surrogates of the entire pulmonary vascular architecture as used in this analysis.

We found no significant associations between PA size and important post-OHT outcomes across the entire population. Patients with smaller PAs did have a significantly higher peak total bilirubin after OHT but were not otherwise at increased risk of developing acute RV failure. Smaller PA size was also associated with several echocardiographic and catheter measures suggestive of RV dysfunction on post-OHT surveillance. Within the CHD cohort, patients with smaller PAs received longer durations of inhaled nitric oxide post-OHT and had a higher incidence of AKI. While these trends may suggest RV dysfunction, there was no significant association between PA size and acute clinical RV failure [3, 13, 16]. Our CHD cohort underwent PA reconstruction during OHT at a higher rate than reported by Chen et al. (78% vs 61%). The primary endpoints in that study were early and late mortality; only two patients in our study died within 30 days of OHT so we did not formally analyze mortality [19]. Despite these findings, we still feel some form of angiography is clinically indicated in patients with single ventricle physiology and those with complex CHD with PA involvement to facilitate sternal re-entry and help guide surgical planning for potential PA reconstruction.

### Limitations

Our study is limited by its retrospective, single center design. Generalizability may be limited by institutional variation in the OHT selection process as well as surgical volume and expertise. Patients with unfavorable pulmonary anatomy may not have been listed for OHT and thus not included in the study. Our sample size was small which resulted in large confidence intervals and relatively weak statistical power; a larger multicenter study would be needed to support or refute our findings. Outcome differences between CHD and non-CHD cohorts may be secondary to longer cross-clamp times and generally more complex transplant surgical procedures in the CHD cohort rather than PA size alone. Similarly, while we used a clinical definition of acute post-OHT RV failure derived from prior studies in similar populations, we acknowledge that this cohort has inherent pre-OHT end-organ dysfunction along with multiple peri-operative threats which may impact our ability to detect meaningful changes in end-organ function post-OHT [3, 13, 16].

With regards to assessment of PA size, we acknowledge that the LLI is likely a more accurate surrogate for the overall pulmonary vascular bed as the lower lobe branches are less subject to surgical revision than the proximal branches assessed by the NI. We observed a reasonable discrepancy in PA measurements for each patient across the different modalities and suggest that CTA represents the gold standard, particularly given the technical limitations of measuring PAs by echocardiography. Previous studies have demonstrated that cardiovascular structures may measure significantly smaller by echocardiography than by CTA, with the proposed explanation that the 3-dimensional data obtained by CTA is more accurate than the 2 dimensions captured by echocardiogram [20].

## Conclusion

Although PA size was associated with several trends suggestive of RV dysfunction in our population, it was not independently predictive of acute RV failure or other important post-OHT outcomes. These negative findings seem to be in line with recent surgical literature in related CHD patient populations. We propose that evolution in OHT surgical technique as guided by pre-operative imaging may mitigate the impact of smaller proximal PA size. Our cohort included patients with NI below 100 mm^2^/m^2^ who underwent successful OHT. From our experience, there does not appear to be a contraindication to OHT in patients with small PAs who are otherwise good candidates. Given the rarity of outcomes in our cohort, a more conclusive determination of the impact of PA size may require follow up multicenter studies to investigate a larger population.

## Data Availability

No datasets were generated or analysed during the current study.

## Abbreviations

AKI: Acute kidney injury
BSA: Body surface area
CHD: Congenital heart disease
CTA: Computed tomography angiography
DCM: Dilated cardiomyopathy
ICU: Intensive care unit
LOS: Length of stay
LLI: Lower lobe index
NI: Nakata index
OHT: Orthotopic heart transplant
PA: Pulmonary artery
POD: Post-operative day
PVR: Pulmonary vascular resistance
RCM: Restrictive cardiomyopathy
RV: Right ventricle
VIS: Vasoactive-inotropic score
